# Pharmacokinetics and Tissue Distribution of Alnustone in Rats after Intravenous Administration by Liquid Chromatography-Mass Spectrometry

**DOI:** 10.3390/molecules24173183

**Published:** 2019-09-02

**Authors:** Yang Song, Yu Zhou, Xiao-Ting Yan, Jing-Bo Bi, Xin Qiu, Yu Bian, Ke-Fei Wang, Yuan Zhang, Xue-Song Feng

**Affiliations:** 1 School of Pharmacy, China Medical University, Shenyang 110122, China; 2 Department of Pharmacy, National Cancer Center/National Clinical Research Center for Cancer, Chinese Academy of Medical Sciences and Peking Union Medical College, Beijing 100021, China

**Keywords:** pharmacokinetics, tissue distribution, alnustone, rats, LC-MS/MS

## Abstract

Alnustone, a nonphenolic diarylheptanoid, first isolated from *Alnus pendula* (Betulaceae), has recently received a great deal of attention due to its various beneficial pharmacological effects. However, its pharmacokinetic profile in vivo remains unclear. The purpose of this study is to establish a fast and sensitive quantification method of alnustone using liquid chromatography tandem mass spectrometry (LC-MS/MS) and evaluate the pharmacokinetic and tissue distribution profiles of alnustone in rats. The sample was precipitated with acetonitrile with 0.5% formic acid and separated on BEH C_18_ Column. The mobile phase was composed of 0.1% formic acid in water and methanol at a flow rate of 0.3 mL/min. Alnustone and the internal standard (caffeine) were quantitatively monitored with precursor-to-product ion transitions of *m*/*z* 262.9→105.2 and m/z 195.2→138.0, respectively. The calibration curve for alnustone was linear from 1 to 2000 ng/mL. The intra- and inter-day assay precision (*RSD*) ranged from 1.1–9.0 % to 3.3–8.6%, respectively and the intra- and inter-day assay accuracy (*RE*) was between −8.2–9.7% and −10.3–9.9%, respectively. The validated method was successfully applied to the pharmacokinetic studies of alnustone in rats. After single-dose intravenous administration of alnustone (5 mg/kg), the mean peak plasma concentration (C_max_) value was 7066.36 ± 820.62 ng/mL, and the mean area under the concentration-time curve (AUC_0–t_) value was 6009.79 ± 567.30 ng/mL∙h. Our results demonstrated that the residence time of alnustone in vivo was not long and it eliminated quickly from the rat plasma. Meanwhile, the drug is mainly distributed in tissues with large blood flow, and the lung and liver might be the target organs for alnustone efficacy. The study will provide information for further application of alnustone.

## 1. Introduction

Alnustone, a nonphenolic diarylheptanoid with a typical chemical structure of an aryl-C7-aryl skeleton, was first isolated from the male flower of *Alnus pendula* (Betulaceae) [1,2,3]. Then, it was also found in the seeds of *Alpinia katsumadai* Hayata (Zingiberaceae) [4,5,6,7,8,9,10], the rhizomes of *Curcuma xanthorrhiza* Roxb [11,12] and *Curcuma comosa* Roxb (Zingiberaceae) [13]. It is reported that diphenylheptanes have a wide range of pharmacological activities, such as anti-inflammatory, hepatoprotective, antioxidant and anti-tumor effects [14,15,16]. Diphenylhexane natural drugs account for 5% of the market share of neurosinosidase inhibitors [17]. Alnustone reportedly exhibits a variety of activities, including antihepatotoxic [18], anti-inflammatory [11], antibacterial [4], antiemetic [5,8,9] and weak estrogenic [13]. Recently, Grienke et al. revealed that alnustone showed neuraminidase inhibitory activity, and it was concluded that the compound may be employed as an antiviral agent [6]. Additionally, the chemical compounds from *Alpinia katsumadai* Hayata seeds have been evaluated for their antitumor activities in vitro. Among the isolated compounds, alnustone was found to exhibit significant antitumor activity against the Bel-7402 (human hepatocellular carcinoma cells) and LO-2 (human normal liver cells) cell lines [19]. As a good drug candidate, several methods have been developed to prepare alnustone, including the purification from plants [16] and synthesis through an organic method [20,21].

Although the bioactivities have been investigated extensively, a few analytical methods have been reported. Currently, there are only three articles concerning the analytical methods for the determination of alnustone in natural medical plants or Chinese patent medicine, including HPLC [22,23], semi-preparative liquid chromatography [24]. As far as the authors know, the pharmacokinetics of this compound still remains unknown. It is generally accepted that the study of pharmacokinetics and tissue distribution plays an important role in the drug development because it helps to predict and explain the various issues associated with drug efficacy and toxicity [25,26]. Therefore, it is necessary to establish an effective method to investigate the pharmacokinetic characteristics of alnustone so as to better understand its mechanism of action.

In the present study, a liquid chromatography tandem mass spectrometry (LC-MS/MS) method was developed and validated for the determination of alnustone in rat plasma. The pharmacokinetic behavior and tissue distribution of the intravenous injection of alnustone in rats was subsequently investigated by this method. To the authors’ knowledge, this is the first report on the pharmacokinetics of alnustone.

## 2. Results and Discussion

### 2.1. Method Establishment

#### 2.1.1. Optimization of LC–MS/MS Conditions

**Figure 1:**
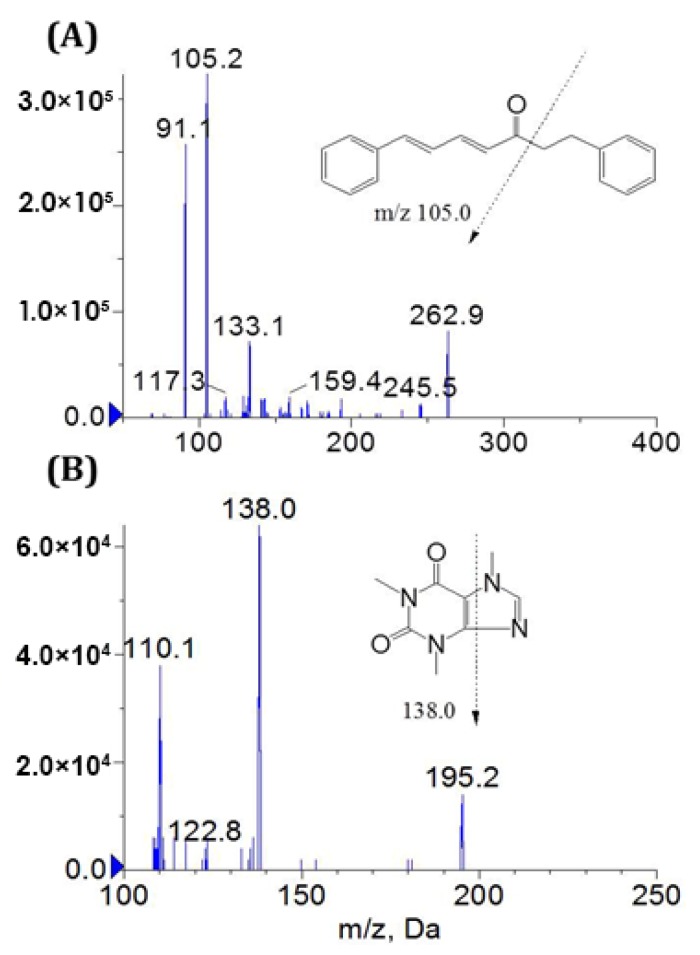
Full-scan product ion spectra of [M + H]^+^ for alnustone (**A**) and caffeine (IS, **B**).

Since the rat plasma contains complex endogenous components, it is necessary to establish a sensitive, rapid and effective method to quantitatively determine the concentration of nanogram alnustone with caffeine (IS) in rat plasma. In this experiment, the electrospray ionization (ESI) source in the positive and negative ion mode was compared. The results showed that the targeted substances can be observed to be more stable and more responsive in the positive ion mode. In order to improve the specificity of the detection method, MS/MS ion transition was monitored in multiple-reaction monitoring (MRM) mode. For each analyte, a precursor ion and two MRM transitions were established, monitoring the more abundant product ion (quantifier ion) for the quantification and the less abundant product ion (qualifier ion) for verification. Figure 1 showed the product ion mass spectrum of alustone and IS with their chemical structures and the chemical bond breaking positions. After giving certain collision energy, the richest and the most stable product ions for alnustone and IS were monitored at *m*/*z* 105.2 and *m*/*z* 138.0, respectively. The MRM transitions for alnustone and IS were *m*/*z* 262.9→105.2 and *m*/*z* 195.2→138.0, which were employed for quantitative analysis. The qualifier ions for alnustone and IS were set at *m*/*z* 133.1 and *m*/*z* 110.1, respectively. The parameters were improved by the maximum intensity observed for product ions, including the ionspray voltage, the collision cell exit potential, the entrance potential, the declustering potential (DP) and the collision energy (CE). Among them, the DP of alnustone and IS were set at 91 V and 92 V; the CE were 18 eV and 30 eV, respectively.

The optimization of chromatographic conditions included the selection of a suitable mobile phase and chromatographic column. The results showed that methanol as the organic phase gave a better peak shape and lower background noise for the analytes than acetonitrile. In addition, in order to improve the ion response and the peak shape of analytes, this study selected and tested two types of ion additives, 5 mmol ammonium acetate and 0.1% fomic acid. The results showed that although the former significantly improved the peak shape, the excessive acid induced-ion suppression decreased the signal of the tested compounds, while the latter was acceptable in improving the peak shape and signal intensity. In addition, the gradient eluting mode was optimized to make the tested compounds completely separated and have good peak shapes, and the interference between the analyte and the IS was avoided. The modified gradient elution conditions were proved to meet the analytical requirements in terms of column equilibrium and carry over. When the flow rate of the mobile phase was 0.3 mL/min, ACQUITY UPLC BEH C_18_ column was used, and the analysis time of a single sample was less than 6.5 min.

#### 2.1.2. Optimization of Pretreatment Method

To optimize the pretreatment method of the simulated biological samples, a simple and rapid protein precipitation method was attempted as the purification and enrichment method of biological samples. The extraction recovery and matrix effect of alnustone and IS with acetonitrile or methanol as the precipitation reagent were investigated. Acetonitrile was selected as the precipitator due to the higher extraction efficiency and better repeatability. Moreover, the extraction efficiency increased when the acetonitrile contained 0.5% formic acid. The different acetonitrile plasma volume ratios (3:1, 4:1 and 5:1, *v*:*v*) were determined. At this ratio of 3:1, the plasma protein turned out to be completely precipitated. In conclusion, the direct protein precipitation method was simple and effective, with high extraction recovery and small background interference.

#### 2.1.3. The Selection of Internal Standard

It is well known that the use of internal standards can improve the accuracy and precision of a quantitative analysis and the stability of the method. Generally speaking, there are two types of internal standards, namely, the structural analogues and the stable isotope labeling (SIL-IS). Although SIL-IS has good performance, it is expensive and difficult to get. In this case, the structural analogue is usually applied as the internal standard. In this study, based on the existing experimental conditions, none of the alnstrone structural analogs were available. Therefore, several non-structural analogues were tested as the internal standard, including caffeine, phenoxetine and paracetamol. Among them, caffeine had similar chromatographic behavior and ionization efficiency with the analyte alnustone, and the extraction recovery and matrix effect met the requirements of the determination. Therefore, caffeine was selected as the internal standard.

### 2.2. Method Validation

#### 2.2.1. Selectivity and Carry-Over

**Figure 2:**
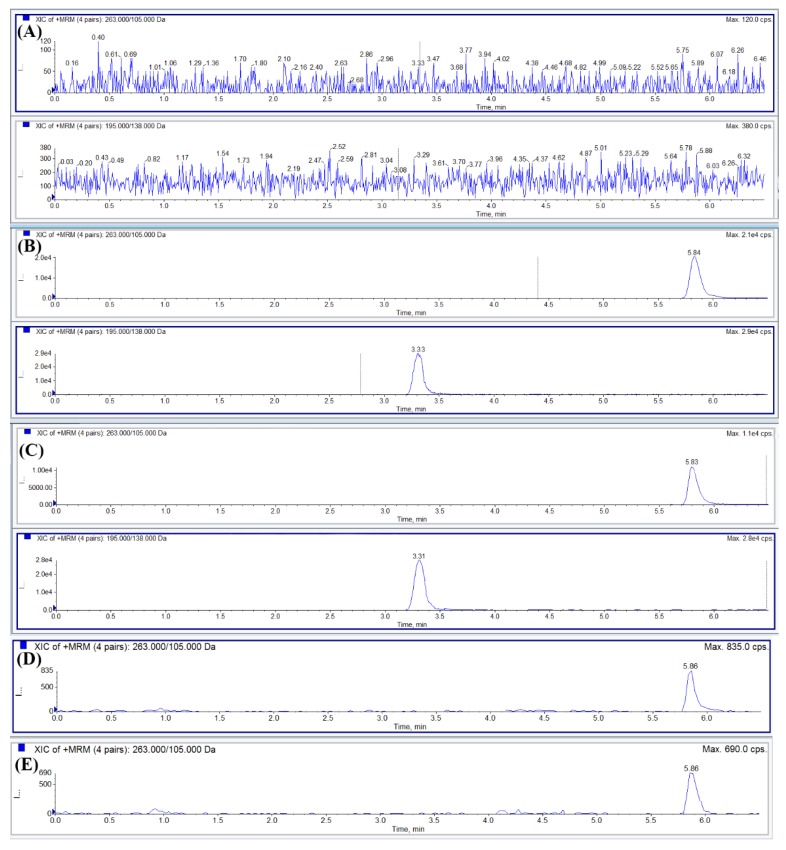
Representative chromatograms of: (**A**) alnustone (5.84 min) and IS (3.33 min) obtained by the extraction of blank plasma; (**B**) blank plasma spiked with alnustone and IS; (**C**) plasma sample from a rat 2h after an intravenous administration of alnustone (5 mg/kg) to rats; (**D**) blank liver tissue homogenates spiked with alnustone at LLOQ; (**E**) blank plasma spiked with alnustone at LLOQ.

No interference peaks were observed at the retention time of alnustone and IS in the chromatograms of six batches of the blank plasma samples. Figure 2 illustrated representative chromatograms of the blank samples, the blank samples spiked with alnustone at the lower limit of the quantification (LLOQ) and IS, and the samples after alnustone administration. The response of the blank samples was compared with the LLOQ samples. The results suggested that there was no obvious endogenous interference in the determination of alnustone and IS.

In the chromatograms of blank samples injected immediately after the HQC samples were injected, no peak was observed at the retention time of alnustone and IS, indicating that the carry-over effect in the sample analysis could be ignored.

#### 2.2.2. Linearity

For blood samples and tissue samples, the linear calibration curves were calculated by a linear regression model. The linear equations of the calibration curves, the linear regression coefficients and the linear ranges were given in Table 1. Each coefficient of determination (*r*) in all validation batches were all greater than 0.99.

#### 2.2.3. Accuracy and Precision

Table 2. showed the results of intra- and inter-day accuracy and the precision of alnustone in the biological matrices of rats. The *RE* values of intra- and inter-day accuracies ranged from −10.3% to 9.9%, and the *RSD* values of the precision assays were all not more than 9.0%, indicating that the precision and accuracy results of this method were within the acceptable criteria, proving that this method was reliable and reproducible to quantitatively analyze alnustone in rat biological samples.

#### 2.2.4. Recovery and Matrix Effect

As shown in Table 3, the extraction recoveries were within the range of 86.3–110.4%, indicating that the optimized pretreatment method of direct protein precipitation could offer good extraction efficiency for alnustone in these matrices. The matrix effects of alnustone ranged from 89.5% to 114.4%, showing that the matrix had a little co-eluting endogenous substance that could influence the ionization of alnustone.

#### 2.2.5. Stability

The results of the stability studies were shown in Table 4, indicating that there was no stability issue occurred.

### 2.3. Pharmacokinetic Study and Tissue Distribution

**Figure 3:**
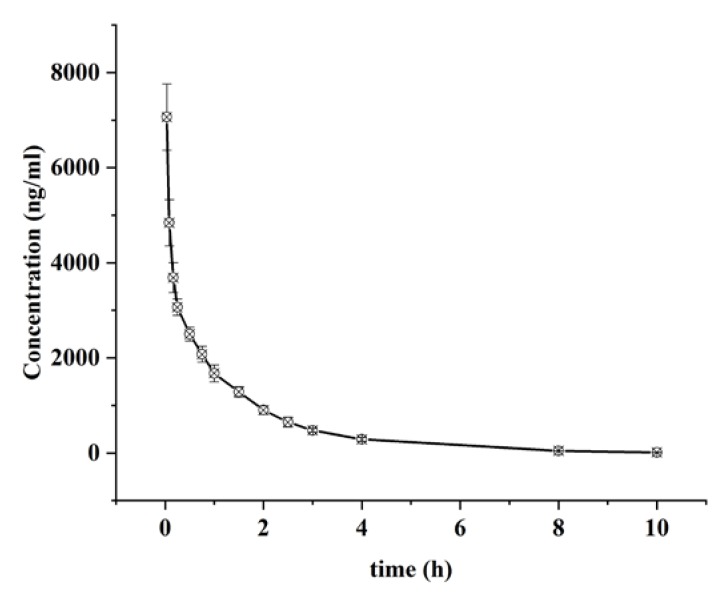
The mean plasma concentration-time curves of alnustone after the intravenous administration of alnustone at a dose of 5 mg/kg (*n* = 12, mean ± SD).

The validated LC-ESI-MS/MS method was successfully applied to a pharmacokinetics and tissue distribution of alnustone after the intravenous administration at a single dose of 5 mg/kg. The intravenous administration dose was generally determined based on 1/20–1/50 of the LD50 value or 1/2–1/3 of the maximum tolerated dose of the reference MTD [27,28,29]. The LD50 obtained by our previous pharmacological experiment was 200 mg/kg and therefore the selected i.v. dose was 5 mg/kg. Meanwhile, it was reported that the structural analog of alnustone was given to the rat i.v. at a dose of 4.5 mg/kg for a pharmacokinetic study [30]. The dosage of another piece of literature was 5.45 mg/kg for the pharmacological activity study [31]. Above all, the dosage of the drug administration in our pharmacokinetics experiment was determined to be 5 mg/kg. Figure 3 showed the mean plasma concentration-time profile of alnustone after administration**.** Table 5 summarized the pharmacokinetic parameters based on the non-compartmental method. Upon intravenous administration of 5 mg/kg alnustone, the *T_max_* was 2 min and the *C_max_* was 7066.36 ± 820.62 ng/mL, indicating that alnustone could be quickly detected in the plasma. The pharmacokinetic results have demonstrated the area under the curve up to 10 h (*AUC_0−t_*) and the infinite time (*AUC_0−∞_*) of 6009.79 ± 567.30 and 6032.45 ± 472.50 ng/mL∙h, respectively. The total mean residence time up to 10 h (*MRT_0-t_*) was found to be 1.60 ± 0.22 h. Alnustone had a relatively short *t_1/2_* of 1.31 ± 0.19 h, an apparent *V_d_* of 1.57 ± 0.18 L/kg, and *CL* of 0.83 ± 0.09 L/h/kg. The short elimination half-life of alnustone in vivo after a single administration indicated that alnustone would not tend to accumulate in rat plasma during long-term use.

**Figure 4:**
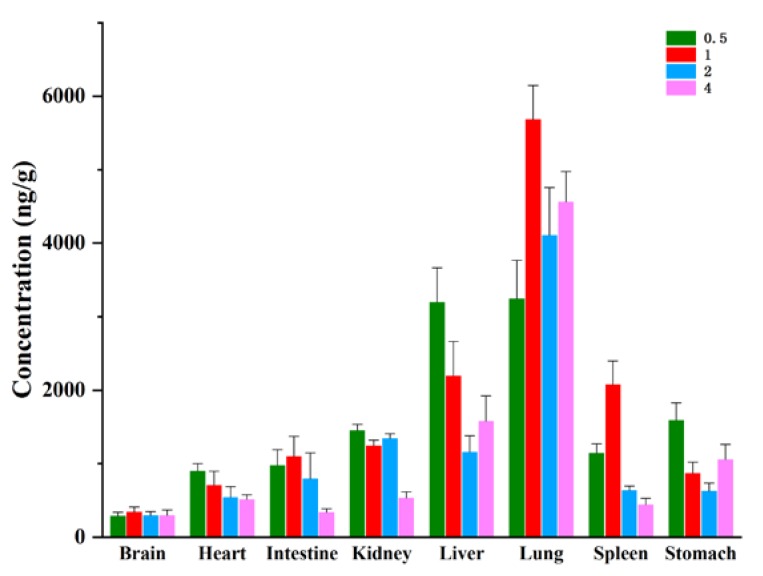
The mean concentration of alnustone in different tissues (ng/g) at 0.5, 1, 2 and 4 h after an intravenous administration of 5 mg/kg alnustone to the rats (Mean ± SD, *n* = 5).

The changing trend of alnustone concentration in different tissues at different time points of 0.5, 1, 2 and 4 h after administration was investigated. Figure 4 revealed that alnustone distributed rapidly and widely in different tissues. In the tissues of brain, intestine, lung, and spleen, the maximum concentration appeared at 1 h, while in heart, kidney, liver and stomach, the maximum concentration of alnustone appeared earlier at 0.5 h to 1h. Among all the tissues, the highest mean concentration was found in the lung (5685.40 ng/g), followed by the liver (3199.49 ng/g), spleen (2077.14 ng/g), stomach (1588.90 ng/g), kidney (1450.43 ng/g), intestine (1099.14 ng/g), heart (900.97 ng/g), and brain (344.27 ng/g). The high level in the lung and liver might be related to the bioaccumulation of alnustone in these two organs. In the liver, an increase of drug exposure was observed at 4 h. Similar situations also occurred in the lung and stomach. The most possible reason was that alnustone might be reabsorbed in these organs. Alustone is fat-soluble, so it might bind to the fat after the first absorption. When the drug reached a certain accumulation in the fat, it would be reabsorbed into the liver, lung and stomach, which have good affinity with the drug. In addition, hepatointestinal circulation may also lead to an increase in the liver at 4 h. Thus, in the liver, lung and stomach, alustone maintained a high concentration and underwent a slow elimination and accumulation. They are highly likely to be the target organs for the efficacy of alnustone, which needs to be studied systematically in the future. In addition, the metabolic clearance rate of alnustone in different tissues was quite different. For example, in the spleen, intestine, and kidney, the elimination rate of alnustone was relatively fast. For 4 h after administration, the concentration of alnustone in these tissues had decreased to less than half of the corresponding maximum concentration. In the other tissues, the elimination rate of alnustone was relatively slow, and the tissue concentrations were either kept at the same level during the measured period or even higher within 4 h after administration. In contrast, due to the rapid elimination and less drug accumulation, alnustone was unlikely to cause drug accumulation and immunological side effects in the spleen, intestine, and kidney. At the same time, the concentration of alnustone in the brain did not change with time, and reached a certain level (567.0 ng/mL, 4 h), suggesting that it could enter the brain through the blood-brain barrier. The tissue distribution experiment provided a reference for the study of the distribution characteristics of drugs in vivo and also provided a theoretical basis for the development of drugs in the later stage.

The tissue distribution characteristics of alnustone were determined at 4 h after i.v. administration of alnustone at 5 mg/kg. The tissue to plasma partition coefficients (Kp) shows an upward trend in 4 h. The Kp values of alnustone in iv administration are summarized in Table 6. The highest Kp was observed in the lung (15.84 ± 1.33), followed by that in the liver (5.48 ± 0.66), stomach (3.66 ± 0.27) tissue, and kidney (1.86 ± 0.18). The highest Kp values and concentrations of alnustone in the lung and liver indicated the possible bioaccumulation of alnustone.

## 3. Materials and Methods

### 3.1. Chemicals and Reagents

Alnustone and caffeine (purity over 98%) were provided by Target Molecule Corp. (TargetMol) (Boston, MA, USA). Acetonitrile and methanol, both are LC-MS-grade, were purchased from Merck KGaA Company (Darmstadt, Germany). The formic acid (HCOOH, HPLC-grade) was obtained from Dikma Technologies Inc. (Lake Forest, CA 92630, USA). The purified water was provided using a Millipore Milli-Q system (Millipore, Bedford, MA, USA).

### 3.2. Instrumentations

The UHPLC-MS/MS method was performed on an Agilent series 1290 UHPLC system (Agilent Technologies, Santa Clara, CA, USA), which was coupled to an AB 3500 triple quadrupole mass spectrometer (AB Sciex, Ontario, ON, Canada) with an electrospray ionization (ESI) source. The separation process was performed on an ACQUITY UPLC BEH C_18_ Column (100 mm × 2.1 mm, 1.7 μm, Agilent Technologies, Santa Clara, CA, USA).

### 3.3. Animals

Further, thirty two Sprague-Dawley rats (male, 200–220 g body weight) were purchased from the Experimental Animal Research Center, China Medical University, China. The protocol for this study (protocol number # CMU2019194) was approved by the Institutional Animal Care and Use Committee at China Medical University. The study complied with guidelines for the Care and Use of Laboratory Animals (published by the National Institutes of Health, NIH publication no. 85–23).

### 3.4. Preparation of Calibration Standards and Quality Control Samples

A 10.0 mg of alnustone was dissolved in 10.0 mL of methanol to give the stock solution with the concentration of 1.0 mg/mL. The working solutions were prepared by serial dilution of the stock solution with the initial mobile phase (methanol—0.1% fomic acid water, 20:80, *v*/*v*). A 10.0 mg of caffeine (IS) was dissolved in 10.0 mL of methanol to give the IS stock solution with the concentration of 1.0 mg/mL. All of the solutions were stored at 4 °C before use.

The calibration standards were prepared by spiking a certain volume of blank plasma or blank tissue homogenates with appropriate amounts of working solutions to yield a final concentration range from 1 to 2000 ng/mL. The effective concentrations of alnustone were 1, 5, 10, 40, 160, 200, 800, 2000 ng/mL for the plasma samples and tissue homogenates samples (heart, liver, spleen, lung, kidney, brain, stomach, intestine). The quality control (QC) samples were prepared in the same manner at three concentration levels (low, mid, high) of 5, 100, 1600 ng/mL for the plasma and tissue homogenates.

### 3.5. Preparation of Plasma and Tissues Samples

In this study, a direct protein precipitation method was applied to extract alnustone and IS from the biological matrix. An aliquot 100 μL of plasma, 50 μL of IS solution (1 μg/mL) and 200 μL of precipitate agent acetonitrile with 0.5% formic acid was added into a 1.5 mL Eppendorf tube. The mixture was vortex-mixed for 30 s at room temperature, and centrifuged at 12,000× *g* for 10 min. Then, a 200 μL aliquot of the supernatant was carefully removed and transferred to a new 1.5 mL Eppendorf tube and evaporated to dryness at 40 °C under a slight stream of nitrogen. The residuals were reconstituted in 100 μL of methanol—0.1% fomic acid water (20:80, *v*/*v*) by vortex mixing for 30 s. After centrifuging at 12,000× *g* for 10 min, a 10 μL aliquot of the supernatant was used for the UHPLC-MS/MS analysis.

The rat was sacrificed quickly by decapitation on the ice. The various tissues (heart, liver, spleen, lung, kidney, brain, stomach, intestine) were harvested and rinsed with ice-cold 0.9% NaCl to remove the superficial blood. After being blotted dry with filter paper, each tissue sample was weighed on ice and physiological saline added (1:2, *w*/*v*) to homogenize. Then, a 100 μL (equivalent to 50 mg) of tissue homogenate was taken and processed using the same method as the plasma samples processing method as shown in the above steps.

### 3.6. Chromatographic and Mass Conditions

The mobile phase consisted of methanol and 0.1% formic acid water using a gradient dilution at a flow rate of 0.3 mL/min. The column temperature was maintained at 30 °C. Alnustone was quantitatively determined with MRM in the positive ion mode, and nitrogen was used to assist nebulization in the ESI source. The MS parameters were set as follows: The ionspray voltage (IS) 5500 V; nebulizer gas (gas 1) 19 arbitrary units; curtain gas (CUR) 10 arbitrary units; collision cell exit potential (CXP) 7.0 V; entrance potential (EP) 10.0 V. The optimization of the MS transitions using the multiple-reaction monitoring (MRM) mode were accomplished as alnustone *m*/*z* 262.9→105.2 and IS (caffeine) *m*/*z* 195.2→138.0, respectively.

### 3.7. Method Validation

The method was fully validated according to the Bioanalytical Method Validation Guidance for Industry (FDA, 2018) [32].

#### 3.7.1. Selectivity

Selectivity was investigated by comparing chromatograms of blank rat plasma obtained from six individual rats with those of the corresponding standard biosample spiked with alnustone at LLOQ and IS and the actual plasma sample 2h after an intravenous administration of alnustone (5 mg/kg).

#### 3.7.2. Linearity and LLOQ

The calibration curves for alnustone in the plasma or tissue homogenates were generated by plotting the peak area ratios (*y*) of alnustone to IS versus those nominal concentrations (*x*) in standard plasma or tissue homogenates using weighted (1/*x^2^*) least squares linear regression. The LLOQ was defined as the lowest concentration of the calibration curve at which the precision did not exceed 20%, the accuracy was within ±20% and the signal-to-noise ratio (S/N) was at least 10.

#### 3.7.3. Accuracy and Precision

The intra- and inter-day accuracy and precision were assessed by analyzing QC samples at three levels of alnustone (5, 100, 1600 ng/mL for plasma and tissue homogenates) on three consecutive days with five replicates at each concentration. The accuracy and precision were depicted as the relative error (*RE*) and the relative standard deviation (*RSD*), respectively. The *RE* was within ±15% and the *RSD* could not exceed 15%.

#### 3.7.4. Recovery and Matrix Effect

The recovery was determined by comparing the peak areas of alnustone or IS in processed QC samples with the mean peak areas of alnustone or IS in the spike-after-extraction samples (blank plasma or tissue homogenates extracted then spiked with QC standards). (*n* = 5)

The matrix effect was evaluated by comparing the peak areas of alnustone or IS in the spike-after-extraction samples (blank plasma or tissue homogenates extracted then spiked with QC standards) with the mean peak areas of alnustone or IS dissolved with the mobile phase at high, medium and low levels, respectively. (*n* = 5). It was generally considered that the matrix effect was obvious if the ratio was <85% or >115%.

#### 3.7.5. Stability

The stability tests involved the following four conditions. The short-term stability study was examined by analyzing samples at room temperature for 4 h. The long-term stability study was performed by analyzing samples stored at −75 °C for 1 month. For the freeze-thaw stability study, the samples were analyzed after three freeze/thaw cycles (−75 °C to 25 °C). The post-preparative storage stability was assessed by analyzing the samples left in autosampler vials at 4 °C for 24 h. The stability analysis was performed using three aliquots of each QC samples at three different concentrations. The samples were considered stable if the assay values were within the acceptable limits of accuracy (±15% *RE*) and precision (≤15% *RSD*).

### 3.8. Drug Administration and Sampling

This study was approved by the Animals Experimental Ethical Committee of China Medical University (Liaoning, China). Alnustone was dissolved in 0.5% (*v*/*v*) DMSO saline to give the injection solution at the dose of 5 mg/kg for intravenous administration.

For the pharmacokinetic study, 12 SD rats fasted for 12 h with free access to water prior to administration. After the rats were tail intravenously administrated of alnustone (5 mg/kg), a blood sample (0.4 mL) was collected from the orbital vein into heparinized tubes at appropriate time intervals (2 min, 5 min, 10 min, 15 min, 30 min, 45 min, 1 h, 1.5 h, 2 h, 2.5 h, 3 h, 4 h, 8 h, 10 h). The blank plasma samples were prepared before dosing. All blood samples were immediately centrifuged at 12,000× *g* for 10 min and stored at –75 °C until analysis.

For the tissue distribution study, 20 SD rats were randomly divided into four groups (5 rats for each time point) and fasted for 12 h with free access to water prior to administration. After the rats were tail intravenously administrated of alnustone (5 mg/kg), the tissue specimens (heart, liver, spleen, lung, kidney, brain, stomach and intestine) were collected at 0.5, 1, 2, and 4 h post-dosing, respectively. The blank tissues were prepared separately using drug-free SD rats. The tissue harvesting and homogenizing method were given in Section 3.5. For the plasma and tissue homogenate sample preparation method, also see Section 3.5.

### 3.9. Statistical Analysis

The pharmacokinetic parameters were evaluated using DAS 3.2.8 pharmacokinetic program [33].

## 4. Conclusions

In this study, a rapid, simple and sensitive UHPLC–ESI MS/MS method was established and successfully applied to the study of pharmacokinetics and tissue distribution of alnustone after a single intravenous administration with 5 mg/kg alnustone in rats. The method has been validated for the first time in this paper and proved to be able to detect a low concentration of 1 ng/mL for alnustone with one direct protein precipitation method. The elimination half-life of alnustone was short implying the residence time of alnustone in vivo was not long and it eliminated quickly from the rat plasma. According to the investigation of tissue distribution, alnustone was mainly distributed in the lung and liver tissues within 1 h, and the drug in each tissue increased slightly at 4 h, which indicated that the drug was mainly distributed in the tissues with large blood flow, and the lung and liver might be the target organs for the curative effect of alnustone. The developed and validated LC-MS/MS method and the pharmacokinetic study of alnustone in the present paper might provide helpful information for its future preclinical applications and theoretical basis for its further exploitation.

## Figures and Tables

**Table 1:** Calibration curves, correlation coefficients, linear ranges and lower limit of the quantification (LLOQ) of alnustone in different biological matrices.

Samples	Calibration Curves	Correlation Coefficients (r)	SES *	SEI ^#^	Linear Ranges (ng/mL)	LLOQs (ng/mL)
Plasma	Y = 0.0256 + 0.00838x	0.996	5.5 × 10^−5^	0.13	1–2000	1
Intestine	Y = 0.022 + 0.00302x	0.991	6.7 × 10^−5^	0.12	1–2000	1
Heart	Y = 0.0174 + 0.00197x	0.992	1 × 10^−4^	0.12	1–2000	1
Liver	Y = 0.063 + 0.00146x	0.993	9.5 × 10^−5^	0.10	1–2000	1
Spleen	Y = 0.00038 + 0.00414x	0.992	1.2 × 10^−4^	0.18	1–2000	1
Lung	Y = 0.0138 + 0.002x	0.995	1.2 × 10^−4^	0.09	1–2000	1
Kidney	Y = 0.0359 + 0.00207x	0.994	8.9 × 10^−5^	0.15	1–2000	1
Stomach	Y = –0.00332 + 0.00194x	0.996	6.2 × 10^−5^	0.22	1–2000	1
Brain	Y = −0.0458 + 0.00287x	0.995	1.4 × 10^−4^	0.17	1–2000	1

* SES—Standard error of the slope (*n* = 6). # SEI—Standard error of the intercept (*n* = 6).

**Table 2:** Intra- and inter-day accuracy and precision of alnustone in the plasma and tissue homogenates of rats (*n* = 5).

Samples	QC Conc. (ng/mL)	Intra-Day		Inter-Day	
		Precision (*RSD*, %)	Accurary (mean%)	Precision (*RSD*, %)	Accurary (mean%)
Plasma	1	7.2	8.8	7.5	6.9
	5	1.1	9.7	5.0	9.9
	100	6.3	−6.5	4.3	−6.2
	1600	5.2	−8.2	8.1	6.9
Heart	1	8.1	−9.7	6.6	−10.7
	5	6.4	8.0	5.7	−10.3
	100	3.6	3.7	7.5	2.7
	1600	8.0	3.7	4.3	3.1
Liver	1	5.7	4.3	5.8	5.0
	5	4.3	7.7	4.1	−6.6
	100	8.9	9.7	5.6	0.6
	1600	9.0	−5.9	6.1	6.6
Spleen	1	9.0	−10.3	7.6	−6.4
	5	7.0	-3.6	5.0	−1.3
	100	7.0	2.2	3.6	5.7
	1600	1.9	8.0	4.1	−6.8
Lung	1	6.6	−8.3	5.8	−5.1
	5	5.7	−6.2	5.3	−1.5
	100	7.5	0.6	6.5	8.1
	1600	5.1	−8.1	6.7	4.3
Kidney	1	4.9	11.2	5.7	10.0
	5	4.2	−6.0	4.4	6.1
	100	6.6	8.0	6.6	1.2
	1600	3.5	−7.2	8.4	−5.3
Brain	1	5.7	8.6	6.1	9.3
	5	7.9	1.3	3.3	9.6
	100	5.9	5.5	8.6	−3.4
	1600	5.9	−4.5	3.3	1.4
Intestine	1	4.9	−7.9	6.8	−4.1
	5	4.1	−1.8	4.3	−6.1
	100	8.0	4.2	5.9	2.6
	1600	7.5	−2.1	6.8	−1.8
Stomach	1	6.2	−8.7	5.3	−5.2
	5	4.9	3.5	3.5	−7.69
	100	2.8	−3.1	7.9	0.8
	1600	7.0	2.8	6.4	−6.3

**Table 3:** The matrix effect and recovery of alnustone in different samples (*n* = 5).

Samples	QC Conc. (ng/mL)	Matrix Effect		Recovery	
		Mean ± SD (%)	RSD (%)	Mean ± SD (%)	RSD (%)
Plasma	1	113.7 ± 6.9	5.2	83.5 ± 5.6	5.4
	5	105.4 ± 4.3	4.1	88.7 ±4.4	5.0
	100	91.3 ± 9.6	10.5	95.7 ± 2.2	2.3
	1600	89.5 ± 4.3	4.8	95.4 ± 3.1	3.3
Heart	1	102.1 ± 5.2	5.0	105.1 ± 4.2	4.3
	5	93.8 ± 4.3	4.6	88.3 ± 2.6	3.0
	100	98.2 ± 7.6	7.7	102.3 ± 3.8	3.7
	1600	109.1 ± 3.7	3.4	98.7 ± 1.6	1.7
Liver	1	116.9 ± 10.3	8.0	85.7 ± 5.6	4.1
	5	114.4 ± 9.9	8.6	91.1 ± 3.2	3.5
	100	97.7 ± 9.1	9.3	95.8 ± 4.3	4.4
	1600	98.7 ± 4.6	4.6	90.1 ± 4.0	4.4
Spleen	1	113.5 ± 4.9	6.5	102.9 ± 6.0	4.7
	5	106.1 ± 5.4	5.1	106.9 ± 4.0	3.7
	100	109.8 ± 5.6	5.1	91.2 ± 3.9	4.3
	1600	112.9 ± 4.3	3.8	86.7 ± 0.4	0.4
Lung	1	112.6 ± 6.3	7.9	90.3 ± 6.7	4.6
	5	103.6 ± 7.6	7.3	92.8 ± 3.1	3.3
	100	91.1 ± 9.6	10.6	94.7 ± 1.3	1.3
	1600	92.0 ± 9.8	10.6	87.6 ± 2.7	3.1
Kidney	1	80.7 ± 8.0	5.6	87.8 ± 6.1	5.0
	5	93.9 ± 7.4	7.9	90.1 ± 2.0	2.3
	100	113.4 ± 3.6	3.2	91.8 ± 2.0	2.2
	1600	109.9 ± 8.1	7.4	97.4 ± 5.4	5.5
Brain	1	113.4 ± 6.0	6.2	90.3 ± 6.0	5.8
	5	105.7 ± 5.5	5.2	95.7 ± 4.2	4.4
	100	99.2 ± 3.0	3.0	92.4 ± 3.6	3.9
	1600	94.3 ± 3.4	3.6	110.4 ± 3.5	3.2
Intestine	1	89.6 ± 6.7	6.1	90.7 ± 5.7	5.8
	5	92.0 ± 2.0	2.2	91.8 ± 4.3	4.7
	100	107.3 ± 8.2	7.7	93.4 ± 3.5	3.7
	1600	90.7 ± 7.7	8.5	96.7 ± 3.1	3.2
Stomach	1	113.5 ± 6.2	6.2	88.6 ± 5.6	5.0
	5	110.6 ± 2.8	2.5	93.0± 2.3	2.5
	100	89.9 ± 4.6	5.1	86.3 ± 4.1	4.8
	1600	91.2 ± 3.1	3.3	87.6 ± 2.9	3.3

**Table 4:** Short-term, post-preparative storage, freeze-thaw and long-term stability of alnustone in different samples (*n* = 5).

Samples	QC Conc. (ng/mL)	Short-Term (at Room Temperature for 4 h)	Autosampler 4 °C for 24 h	Three Freeze-Thraw Cycles	Storage at −75°C for 30 d
Plasma	1	108.5 ± 7.6	95.5 ± 5.0	114.1 ± 5.1	98.9 ± 5.7
	5	112.7 ± 8.8	93.4 ± 4.3	112.7 ± 4.9	99.2 ± 6.5
	100	99.8 ± 5.6	101.2 ± 7.5	93.2 ± 7.1	107.2 ± 6.7
	1600	104.8 ± 5.2	108.4 ± 5.5	98.6 ± 2.3	94.8 ± 5.0
Heart	1	109.6 ± 6.0	88.7 ± 8.6	103.8 ± 5.3	107.5± 6.3
	5	104.3 ± 4.3	91.8 ± 8.7	109.1 ± 4.1	101.0 ± 5.3
	100	101.0± 3.2	89.0 ± 3.4	104.8 ± 6.6	112.4 ± 4.0
	1600	91.8 ± 5.8	90.3 ± 5.2	95.3 ± 9.0	95.6 ± 5.4
Liver	1	98.8 ± 6.8	93.1 ± 5.7	101.5 ± 10.1	111.5 ± 6.9
	5	95.4 ± 5.8	95.3 ± 3.2	98.8 ± 9.9	106.5 ± 10.1
	100	111.0 ± 4.9	101.3 ± 5.6	108.4 ± 7.8	105.4 ± 7.2
	1600	106.4 ± 6.0	94.8 ± 7.7	98.3 ± 6.3	97.7 ± 6.8
Spleen	1	108.5± 6.4	108.0 ± 6.0	101.2 ± 7.4	102.3 ± 4.9
	5	114.7 ± 5.8	101.8 ± 4.3	106.2 ± 6.9	98.1 ± 3.9
	100	107.5 ± 8.9	92.9 ± 6.5	105.3 ± 3.1	114.3 ± 6.6
	1600	104.4 ± 6.1	102.1 ± 9.3	92.9 ± 7.6	104.0 ± 3.2
Lung	1	89.5 ± 7.6	105.8 ± 7.0	108.2 ± 9.5	103.4 ± 8.7
	5	92.4 ± 6.4	103.4 ± 7.1	105.8 ± 8.7	93.0 ± 9.6
	100	106.4 ± 8.6	98.4 ± 5.4	94.4 ± 2.6	101.0 ± 7.5
	1600	91.2 ± 6.8	100.6 ± 9.7	93.8 ± 4.6	92.3 ± 6.3
Kidney	1	107.8 ± 7.5	116.5 ± 8.0	93.7 ± 7.0	89.8 ± 8.1
	5	110.7 ± 6.6	114.1 ± 7.4	95.7 ± 5.7	88.5 ± 6.4
	100	109.7 ± 8.3	108.0 ± 5.7	90.9 ± 7.0	111.0 ± 7.5
	1600	100.7 ± 7.0	95.9 ± 6.0	112.5 ± 5.2	100.5 ± 10.6
Brain	1	110.6 ± 5.8	109.2 ± 6.7	86.5 ± 6.1	103.8 ± 4.1
	5	109.0± 4.4	104.9 ± 7.0	89.2 ± 4.6	108.9 ± 3.8
	100	99.3 ± 7.5	95.4 ± 5.7	98.0 ± 8.1	90.2 ± 7.4
	1600	96.3 ± 7.4	104.2 ± 4.9	110.2 ± 6.8	112.3 ± 8.9
Intestine	1	89.6 ± 8.1	90.0 ± 5.7	113.7 ± 8.7	96.5 ± 5.7
	5	95.8 ± 7.7	90.9 ± 5.0	108.7 ± 9.4	92.6 ± 3.7
	100	95.3 ± 6.7	98.0 ± 4.9	96.9 ± 3.8	114.4 ± 4.9
	1600	105.9 ± 7.1	103.0 ± 8.4	113.6 ± 2.8	107.0 ± 4.3
Stomach	1	95.1 ± 6.8	93.2 ± 8.5	102.2 ± 5.1	107.4 ± 6.2
	5	93.8 ± 5.9	98.8 ± 9.6	93.2 ± 4.3	94.9 ± 7.2
	100	104.4 ± 5.5	104.4 ± 5.5	114.0 ± 4.9	107.0 ± 6.7
	1600	114.9 ± 6.8	107.9 ± 8.7	104.7 ± 5.8	93.8 ± 7.6

**Table 5:** Non-compartmental plasma pharmacokinetic parameters following a single intravenous administration of alnustone (5 mg/kg) to rats (*n* = 12).

Pharmacokinetic Parameters	Dose of i.v. Administration (5 mg/kg)
Cmax (ng/mL)	7066.36 ± 820.62
*t_1/2_* (h)	1.31 ± 0.19
AUC*_0_*_–*t*_ (ng/mL∙h)	6009.79 ± 567.30
AUC*_0–∞_* (ng/mL∙h)	6032.45 ± 472.50
MRT*_0–∞_* (h)	1.60 ± 0.22
CL (L/h/kg)	0.83 ± 0.09
*V_d_* (L/kg)	1.57 ± 0.18

**Table 6:** Plasma to tissue partition coefficients (Kp) of alnustone after i.v. administration of alnustone (5 mg/kg) to the rats (Mean ± SD, *n* = 5).

Time (h)	Heart	Liver	Spleen	Lung	Kidney	Brain	Intestine	Stomach
0.5	0.36 ± 0.06	1.28 ± 0.34	0.46 ± 0.06	1.30 ± 0.09	0.58 ± 0.07	0.11 ± 0.40	0.39 ± 0.07	0.64 ± 0.14
1	0.42 ± 0.10	1.31 ± 0.18	1.24 ± 0.13	3.39 ± 0.39	0.74 ± 0.11	0.21 ± 0.56	0.66 ± 0.12	0.52 ± 0.11
2	0.59 ± 0.17	1.28 ± 0.29	0.70 ± 0.06	4.55 ± 0.61	1.49 ± 0.27	0. 33 ± 0.10	0.88 ± 0.19	0.70 ± 0.03
4	1.78 ± 0.24	5.48 ± 0.66	1.54 ± 0.32	15.84 ± 1.33	1.86 ± 0.18	1.03 ± 0.08	1.18 ± 0.20	3.66 ± 0.27
